# EpCAM Is a Surface Marker for Enriching Anterior Pituitary Cells From Human Hypothalamic-Pituitary Organoids

**DOI:** 10.3389/fendo.2022.941166

**Published:** 2022-07-12

**Authors:** Yu Kodani, Miho Kawata, Hidetaka Suga, Takatoshi Kasai, Chikafumi Ozone, Mayu Sakakibara, Atsushi Kuwahara, Shiori Taga, Hiroshi Arima, Toshiki Kameyama, Kanako Saito, Akira Nakashima, Hiroshi Nagasaki

**Affiliations:** ^1^ Department of Physiology, School of Medicine, Fujita Health University, Toyoake, Japan; ^2^ Department of Endocrinology and Diabetes, Graduate School of Medicine, Nagoya University, Nagoya, Japan; ^3^ Regenerative & Cellular Medicine Kobe Center, Sumitomo Pharma Co., Ltd., Kobe, Japan; ^4^ Department of Physiological Chemistry, School of Medicine, Fujita Health University, Toyoake, Japan

**Keywords:** human pluripotent stem cells, organoid, hypothalamus, pituitary, cell surface marker, EpCAM

## Abstract

Human stem cell-derived organoid culture enables the *in vitro* analysis of the cellular function in three-dimensional aggregates mimicking native organs, and also provides a valuable source of specific cell types in the human body. We previously established organoid models of the hypothalamic-pituitary (HP) complex using human pluripotent stem cells. Although the models are suitable for investigating developmental and functional HP interactions, we consider that isolated pituitary cells are also useful for basic and translational research on the pituitary gland, such as stem cell biology and regenerative medicine. To develop a method for the purification of pituitary cells in HP organoids, we performed surface marker profiling of organoid cells derived from human induced pluripotent stem cells (iPSCs). Screening of 332 human cell surface markers and a subsequent immunohistochemical analysis identified epithelial cell adhesion molecule (EpCAM) as a surface marker of anterior pituitary cells, as well as their ectodermal precursors. EpCAM was not expressed on hypothalamic lineages; thus, anterior pituitary cells were successfully enriched by magnetic separation of EpCAM^+^ cells from iPSC-derived HP organoids. The enriched pituitary population contained functional corticotrophs and their progenitors; the former responded normally to a corticotropin-releasing hormone stimulus. Our findings would extend the applicability of organoid culture as a novel source of human anterior pituitary cells, including stem/progenitor cells and their endocrine descendants.

## 1 Introduction

The hypothalamus and pituitary gland are a functional unit that integrates the vertebrate endocrine system. Although the hypothalamus regulates the hormonal output of the anterior pituitary *via* a portal system, it also supports pituitary organogenesis by supplying morphogenic factors during the embryonic period (1). We have successfully reproduced this developmental process in a three-dimensional culture of human embryonic stem cells (ESCs) or induced pluripotent stem cells (iPSCs) (2, 3) by modifying our original differentiation protocol for mouse ESCs (4). At the early stage of the three-dimensional differentiation, the oral ectoderm (OE) and hypothalamic neuroepithelium (NE) are generated in contact with each other in a cell aggregate. The OE forms the outer layer of the aggregate and differentiates into anterior pituitary progenitors, which subsequently generate adenohypophyseal hormone-producing cells. The hypothalamic NE constitutes the inner part of the aggregate and produces hypothalamic neuropeptidergic neurons. The presence of the hypothalamic tissue contributes to functional maturation, as well as early differentiation of the adjoining pituitary tissue (3, 5). This hypothalamic-pituitary (HP) organoid is suitable for analyzing HP interactions during human embryogenesis and has recently been utilized as a disease model of congenital pituitary hypoplasia using patient-derived iPSCs (5). This study elegantly demonstrated that a pathogenic mutation in the OTX2 gene impairs the production of fibroblast growth factor 10 in the hypothalamic NE and therefore disrupts pituitary development by increasing apoptosis of pituitary progenitors in the organoids.

On the other hand, isolated pituitary cells from HP organoids would also be useful for various studies on the human endocrine system. For instance, we previously reported that pituitary tissues dissected from HP organoids clearly improve the lifetime and physical activity of hypopituitary mice when transplanted into these animals (2). This therapeutic effect is mainly mediated by adrenocorticotropic hormone (ACTH) secreted from the grafted corticotrophs, suggesting that the enrichment of functional pituitary cells from HP organoids is a promising step toward regenerative medicine for hypopituitarism. Furthermore, the isolation of pituitary lineages from the organoids may offer opportunities to purify human pituitary stem cells. Investigating the regulatory mechanisms of pituitary stem cells plays critical roles in understanding the organogenesis, homeostasis, and regeneration of the normal pituitary gland, as well as the pathophysiology of pituitary disorders, such as tumors (6). Due to the difficulty associated with obtaining human pituitary specimens, numerous studies have focused on stem/progenitor cells in the rodent pituitary gland. Although several methods for the purification of pituitary stem/progenitor cells have been reported (7–19), all methods are designed to use dissected pituitary samples and therefore are not necessarily applicable to HP organoids, which are composed of both hypothalamic and pituitary cells. However, there is no established method that definitively and consistently separates hypothalamic and pituitary cell fractions in the organoids.

To develop such a cell separation method, we aimed to identify the cell surface marker of anterior pituitary cells. To this end, we performed surface marker screening of OE cells in iPSC-derived HP organoids using a panel of antibodies against 332 human cell surface antigens. This analysis identified epithelial cell adhesion molecule (EpCAM; also known as CD326), which is expressed on the OE and its pituitary descendants, but not on hypothalamic lineage cells. EpCAM is a transmembrane glycoprotein widely expressed in normal epithelial tissues, as well as cancer tissues of epithelial origin (20), and its immunoreactivity has also been observed in the human anterior pituitary gland (21–23). We demonstrate that the isolation of EpCAM^+^ cells from HP organoids enables the enrichment of anterior pituitary cells, including ACTH-producing cells as well as a stem/progenitor cell population.

## 2 Materials and Methods

### 2.1 Generation of HP Organoids

Human iPSCs (201B7; RIKEN BRC, #HPS0063) were cultured on mouse embryonic fibroblast feeder cells as described previously (3). Organoid formation was performed under the “modified condition” in Ref (3).. Briefly, dissociated iPSCs were reaggregated in growth factor-free chemically defined medium (gfCDM) supplemented with 10% knockout serum replacement (KSR; Gibco, #10828028) and 20 µM Y-27632 (Wako, #034-24024) using low-cell-adhesion V-bottom 96-well plates (Sumilon PrimeSurface 96V plate; Sumitomo Bakelite, #MS-9096V) (10,000 cells/100 µl/well). The gfCDM consisted of 1:1 Iscove’s modified Dulbecco’s medium (Gibco, #31980030)/Ham’s F12 (Gibco, #31765035), 1% chemically defined lipid concentrate (Gibco, #11905031), 450 µM monothioglycerol (Sigma, #M6145), and 5 mg/ml purified BSA (Sigma, #A3156). The culture start date was defined as day 0. From day 6, 10 nM BMP4 (R&D Systems, #314-BP) and 2 µM SAG (Cayman Chemical, #11914) were added. From day 18, the culture was maintained with a high oxygen concentration (40%) and BMP4 was gradually decreased. Organoids were transferred to non-adherent culture dishes on day 30 and maintained in gfCDM + 20% KSR + 2 µM SAG from day 51 onward.

### 2.2 Surface Marker Screening

On day 47, HP organoids were dissociated with Accumax (Innovative cell technologies, #AM105) to single cells and stained using a LEGENDScreen Human PE Kit (BioLegend, #700001), which contains PE-conjugated 332 surface marker antibodies and 10 isotype controls. This and the following procedures were conducted in low-cell-adhesion V-bottom 96-well plates (1×10^5^ cells/1 surface marker antibody or isotype control/well). After washing *via* centrifugation, cells were fixed and permeabilized using an IntraStain kit (Dako, #K2311) and incubated with an FITC-conjugated mouse anti-cytokeratin monoclonal antibody (1:30; clone CK3-6H5; Miltenyi Biotec, #130-080-101, RRID: AB_244191), 5 µg/ml Hoechst 33342, and HCS CellMask Deep Red Stain (Invitrogen, #H32721) for 20 min at room temperature (RT). Finally, cells were washed in Cell Staining Buffer (BioLegend, #420201) and transferred to 96-well imaging plates (CellCarrier-96; PerkinElmer, #6005550). Fluorescence images from each well were acquired and analyzed using the Opera Phenix high-content imaging system (PerkinElmer, RRID: SCR_021100) to determine the percentage of cell populations automatically (1700–11200 Hoechst^+^ nuclei were detected per well). CellMask signals were used for segmentation of cell areas. Cells stained with isotype controls were used as negative controls to determine positive staining for surface markers.

### 2.3 Immunocytochemistry

Dissociated single cells were incubated with a PE-conjugated mouse anti-EpCAM monoclonal antibody (1:100; clone HEA-125; Miltenyi Biotec, #130-098-115, RRID: AB_2660299) in Cell Staining Buffer for 10 min at 4°C. This monoclonal antibody is widely used for the detection of human EpCAM in flow cytometry and immunohistochemistry and has previously been used for EpCAM staining in the human anterior pituitary gland (21). After washing *via* centrifugation, cells were fixed, permeabilized, and incubated with the anti-cytokeratin antibody and Hoechst 33342, as described above. Stained cells were mounted on glass slides, and their fluorescence images were captured with a DMI6000B microscope (Leica Microsystems).

### 2.4 Immunohistochemistry

HP organoids and other aggregates were fixed with 4% paraformaldehyde for 1 h at RT and cryoprotected in sucrose solutions of increasing concentrations (10, 20, and 30%) for 30 min each, followed by embedding in O.C.T. compound (Sakura, #4583). Serial 10-μm-thick cryosections were prepared with a CM3050S cryostat (Leica Biosystems) and mounted on CREST adhesive slides (Matsunami, #CRE-02). The sections were immersed in blocking buffer (5% normal donkey serum and 0.1% TritonX-100 in PBS) for 30 min at RT and then incubated with primary antibodies in blocking buffer overnight at 4°C. After washing in PBS + 0.05% TritonX-100, the sections were incubated with appropriate secondary antibodies and DAPI in blocking buffer for 1 h at RT. In this step, cytokeratin or EpCAM staining was simultaneously performed with the FITC- or PE-conjugated antibody at the same dilution as in immunocytochemistry when needed. Finally, the sections were washed in PBS, coverslipped with Fluoromount (Diagnostic BioSystems, #K024), and observed with a LSM710 confocal microscope (Carl Zeiss). The primary and secondary antibodies are listed in **Table 1**.

**Table 1:** Primary and secondary antibodies used for immunohistochemistry.

	Host	Dilution	Source; Catalog number	RRID
Primary antibody				
ACTH	Goat	1:200	Santa Cruz Biotechnology; sc-18262	AB_634931
ACTH	Mouse	1:200	Fitzgerald; 10C-CR1096M1	AB_1282437
LHX3	Rabbit	1:3000	Ref (2).	N/A
MAP2	Chicken	1:10000	BioLegend; 822501	AB_2564858
NKX2.1	Rabbit	1:200	Santa Cruz Biotechnology; sc-13040	AB_793532
PITX1	Guinea pig	1:2000	Ref (2).	N/A
RX	Guinea pig	1:2000	TaKaRa; M229	AB_2783559
SOX2	Goat	1:200	Santa Cruz Biotechnology; sc-17320	AB_2286684
Secondary antibody				
Alexa Fluor 488 anti-chicken	Donkey	1:500	Jackson ImmunoResearch; 703-545-155	AB_2340375
Alexa Fluor 488 anti-guinea pig	Donkey	1:500	Abcam; ab150185	AB_2736871
Alexa Fluor 488 anti-goat	Donkey	1:500	Thermo Fisher Scientific; A-11055	AB_2534102
Alexa Fluor 488 anti-mouse	Donkey	1:500	Thermo Fisher Scientific; A-21202	AB_2534088
Alexa Fluor 594 anti-mouse	Donkey	1:500	Thermo Fisher Scientific; A-21203	AB_141633
Alexa Fluor 594 anti-rabbit	Donkey	1:500	Thermo Fisher Scientific; A-21207	AB_141637
Alexa Fluor 647 anti-rabbit	Donkey	1:500	Thermo Fisher Scientific; A-31573	AB_2536183
Cy3 anti-guinea pig	Donkey	1:500	Jackson ImmunoResearch; 706-165-148	AB_2340460

### 2.5 Magnetic-Activated Cell Sorting

On days 90–500, HP organoids were dissociated to single cells as follows. To suppress cell death during dissociation, organoids were pre-cultured with 20 µM Y-27632 at least for 1 h. This concentration of Y-27632 was added to all solutions used in the following steps unless otherwise noted. The pre-treated organoids were minced and incubated for 40 min at 37°C in DMEM/F12 (Wako, #042-30555) containing 0.2% collagenase type I (Wako, #031-17601) and 0.1% BSA with gentle agitation. The fragments were washed in PBS and further incubated for 5–10 min at 37°C in a solution containing 0.25% trypsin/EDTA (Gibco, #25200072), 60 µM Y-27632, and 0.2 mg/ml DNase I (Roche, #11284932001). The fragments were broken into smaller pieces by gentle pipetting, pelleted by centrifugation in gfCDM + 20% KSR, and triturated by vigorous pipetting in gfCDM + 20% KSR + 0.01 mg/ml DNase I. The cell suspension was finally passed through a 70-µm cell strainer (pluriSelect, #43-10070-60).

The dispersed cells were processed using a Dead Cell Removal Kit (Miltenyi Biotec, #130-090-101) with LS Columns (Miltenyi Biotec, #130-042-401). Collected live cells were stained with the PE-conjugated anti-EpCAM antibody (1:100) in magnetic-activated cell sorting (MACS) buffer (0.5% BSA and 2 mM EDTA in PBS) for 10 min at 4°C. After washing *via* centrifugation, the cells were labeled with anti-PE MicroBeads UltraPure (Miltenyi Biotec, #130-105-639) in MACS buffer for 15 min at 4°C. After washing *via* centrifugation, magnetic bead-labeled (EpCAM^+^) and unlabeled (EpCAM^−^) cells were separated using the MACS system with LS Columns. The cells before and after MACS separation were observed using a DMI6000B microscope to quantify the percentage of PE-labeled cells (i.e., EpCAM^+^ cells). The MACS-separated cells were reaggregated in gfCDM + 20% KSR + 30 µM Y-27632 using low-cell-adhesion V-bottom 96-well plates (15,000 cells/200 µl/well) and cultured for 6–19 days prior to the analysis. Half the volume of the medium was changed every 3 days without the addition of Y-27632.

### 2.6 Analysis of ACTH Release

The release of ACTH from EpCAM^+^ and EpCAM^−^ cell aggregates was analyzed at 6–7 days after the MACS experiments, which had been performed using day-415 or -500 organoids. To assess spontaneous ACTH release, six aggregates were transferred to a low protein binding 1.5-ml tube (PROTEOSAVE SS; Sumitomo Bakelite, #MS-4265M) and incubated in 250 µl of HBSS (Wako, #084-08965) for 10 min at 37°C. The supernatant was collected and stored at −80°C. The ACTH concentration in the supernatant was measured using an electrochemiluminescence immunoassay kit (SRL), which is used clinically in Japan. To evaluate the stimulating effect of corticotropin-releasing hormone (CRH) on ACTH release, aggregates were initially incubated in HBSS without CRH, as described above, and then incubated in 250 µl of HBSS containing 1 µg/ml CRH (PEPTIDE INSTITUTE, #4136-s) for 10 min at 37°C. Collected supernatants were analyzed as described above, and the fold change of the ACTH concentration after the addition of CRH was calculated.

### 2.7 Image Analysis

To determine the percentages of cell populations expressing cytokeratin and/or EpCAM in dissociated organoid samples, immunopositive cells, as well as Hoechst^+^ nuclei, were manually quantified in microscopic images using the cell counter plugin for Fiji (RRID: SCR_002285) (24), a distribution of ImageJ (National Institutes of Health). The percentage of the immunopositive cells among total nuclei was then calculated (1100–1500 nuclei counted per sample). To quantify EpCAM^+^ and EpCAM^−^ aggregates containing ACTH^+^ or PITX1^+^/LHX3^+^ cells, immunofluorescence images were acquired from at least three non-adjacent sections per aggregate. In this analysis, aggregates were obtained by four independent MACS experiments that were conducted on days 172, 228, 415, and 500. However, two experimental batches (days 228 and 500) were excluded from the analysis of PITX1^+^/LHX3^+^ cells because of high background or non-specific staining. The percentages of ACTH^+^, PITX1^+^, or LHX3^+^ cells among total DAPI^+^ nuclei were determined for each aggregate using the cell counter plugin for Fiji (200–1100 nuclei counted per aggregate).

### 2.8 Statistical Analysis

Statistical analyses were performed using the R software program (R Project for Statistical Computing, RRID: SCR_001905). Grouped data were presented as the mean ± SEM. Statistical significance was assessed by Welch’s *t* test or a one-sample *t* test. P values of <0.05 were considered statistically significant.

## 3 Results

### 3.1 Identification of EpCAM as a Surface Marker for OE Cells in HP Organoids

**Figure 1:**
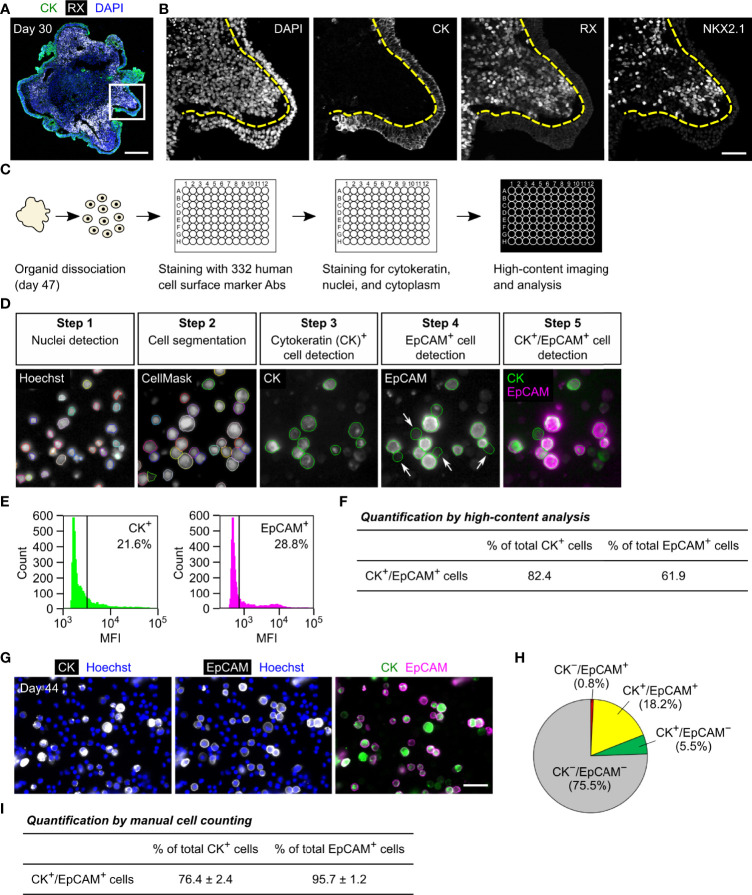
Identification of EpCAM by surface marker screening of OE cells in HP organoids. **(A, B)** Triple immunostaining of a day-30 HP organoid for cytokeratin (CK), RX, and NKX2.1. Nuclei were stained with DAPI. Staining for NKX2.1 is omitted in **(A)**. Magnified views of the boxed region are presented by monochrome images in **(B)**. Dashed lines show the boundary between the cytokeratin^+^ OE layer and RX^+^/NKX2.1^+^ hypothalamic NE. **(C)** The workflow of the screening experiment. Ab: antibody. **(D)** Representative images of the high-content analysis. Cells were stained for nuclei (Hoechst), cytoplasm (CellMask), cytokeratin, and EpCAM. Arrows indicate false-positive EpCAM^+^ cells caused by the contamination of fluorescence signals from contiguous EpCAM^+^ cells. **(E)** The mean fluorescence intensity (MFI) of respective cells in the channels of cytokeratin (left) and EpCAM (right). The vertical lines indicate thresholds that separate positive and negative cell fractions. These threshold settings were consistently used for the high-content analysis. **(F)** The percentage of cytokeratin^+^/EpCAM^+^ double-positive cells among total cytokeratin^+^ or EpCAM^+^ cells. These parameters were automatically quantified for all of the tested surface markers, and therefore EpCAM was only identified as the surface marker for which both parameters exceeded 60%. **(G)** Double immunostaining of suspension cells dissociated from day-44 HP organoids for cytokeratin and EpCAM. **(H)** The percentages of cytokeratin and EpCAM double-positive, single-positive, and double-negative cell populations quantified by manual cell counting. The values represent the mean of four independent experiments on days 44–51. **(I)** The percentage of cytokeratin^+^/EpCAM^+^ double-positive cells among total cytokeratin^+^ or EpCAM^+^ cells. The values were calculated from the same dataset in **(H)**. Scale bars: 200 µm **(A)**, 50 µm **(B, G)**.

For surface marker screening, we used a commercially available LEGENDScreen kit that contains 332 human cell surface marker antibodies. For staining with these antibodies, organoid cells were dissociated using an Accutase-based enzyme mixture (Accumax) based on the manufacturer’s instructions to minimize degradation of surface antigens. In iPSC-derived HP organoids, the OE and hypothalamic NE are differentiated after 1 month of culture (**Figures 1A, B**) and the OE then generates pituitary progenitors and ACTH cells in a stepwise manner (3). Preliminary experiments demonstrated that the organoids are easily dissociated with Accumax to single cells until around day 50. We therefore decided to perform surface marker screening of OE cells using day-47 organoid samples (**Figure 1C**). Dissociated cells were stained for 332 surface markers using the LEGENDScreen kit and then stained for the OE marker cytokeratin, which is homogeneously expressed in the OE layer but completely absent in the RX^+^/NKX2.1^+^ hypothalamic NE in HP organoids (2, 3) (**Figures 1A, B**). We used an anti-cytokeratin antibody that recognizes cytokeratins 7, 8, 18, and 19. Stained cells were analyzed using a high-content imaging system to determine the surface markers that were specifically expressed on cytokeratin^+^ OE cells (**Figures 1D, E**). In this analysis, we defined two selection criteria for candidate surface markers (CSMs): cytokeratin^+^/CSM^+^ double-positive cells must constitute (i) >60% of total cytokeratin^+^ cells and (ii) >60% of total CSM^+^ cells. Among the tested markers, EpCAM was identified as the sole CSM that met both criteria (**Figure 1F**). However, automatic quantification with a high-content imaging system was somewhat influenced by false-positive EpCAM^+^ cells (**Figure 1D**, arrows), which were caused by the contamination of fluorescence signals from contiguous EpCAM^+^ cells.

To further verify the co-expression of cytokeratin and EpCAM, multiple batches of organoids were dissociated on days 44–51 and stained for both molecules (**Figure 1G**). Acquired fluorescence images were subjected to manual cell counting to determine the percentages of double-positive, single-positive, and double-negative cell populations (**Figure 1H**). This analysis showed that cytokeratin^+^/EpCAM^+^ double-positive cells constituted 76% of total cytokeratin^+^ cells and 96% of total EpCAM^+^ cells (**Figure 1I**). Of note, two different monoclonal antibodies against human EpCAM (clones 9C4 and HEA-125) were used in the high-content analysis and manual cell counting, respectively. These antibodies provided qualitatively similar results, supporting the idea that they specifically recognize EpCAM-expressing cells in HP organoids. Thus, our results indicate that EpCAM is a highly specific surface marker for OE cells and that it is not expressed on hypothalamic NE cells at the early stage of HP organoids.

### 3.2 Expression Pattern of EpCAM in HP Organoids

**Figure 2:**
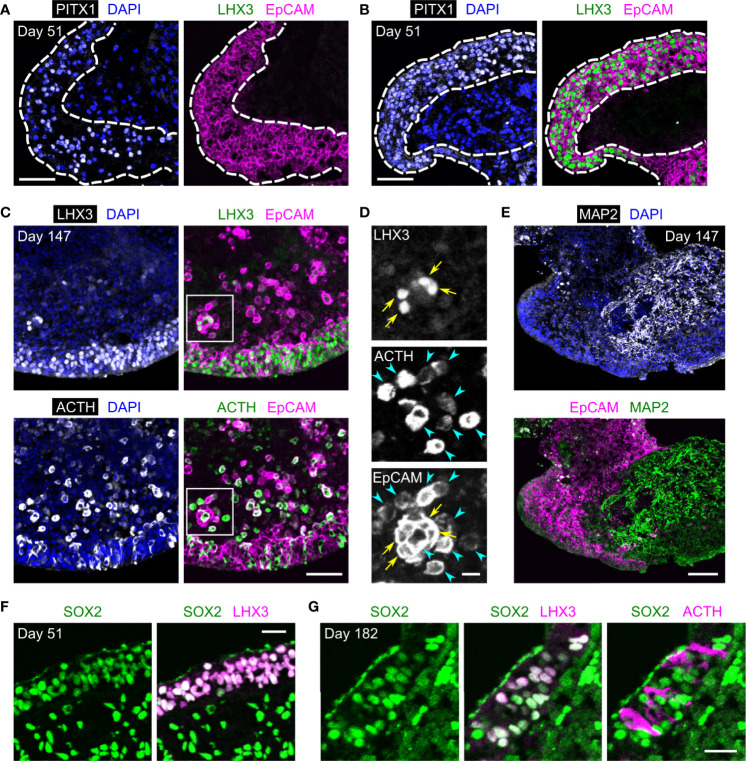
The expression of EpCAM by anterior pituitary cells in HP organoids. **(A, B)** Triple immunostaining of a day-51 HP organoid for PITX1, LHX3, and EpCAM. Nuclei were stained with DAPI. Dashed lines show the surface layers of the organoid consisting of PITX1^+^/LHX3^−^ OE cells **(A)** or PITX1^+^/LHX3^+^ pituitary progenitors **(B)**, both of which express EpCAM. **(C, D)** Triple immunostaining of a day-147 HP organoid for LHX3, ACTH, and EpCAM. Magnified views of the boxed region are presented by monochrome images in **(D)**. EpCAM is co-expressed with LHX3 (arrows) or ACTH (arrowheads). **(E)** Immunofluorescence images of a day-147 HP organoid showing the mutually exclusive expression of MAP2 and EpCAM. **(F)** Double immunostaining of a day-51 HP organoid for SOX2 and LHX3. **(G)** Triple immunostaining of a day-182 HP organoid for SOX2, LHX3, and ACTH. Scale bars: 50 µm **(A–C)**, 10 µm **(D)**, 100 µm **(E)**, 20 µm **(F, G)**.

We previously reported that cytokeratin is expressed in the OE and its pituitary descendants in developing HP organoids (2, 3). To examine whether EpCAM follows this expression pattern, we performed immunohistochemical analyses of HP organoids. OE cells are known to express a specific marker, PITX1, and additionally express LHX3 when committed to anterior pituitary progenitors (2–4). In day-51 organoids, EpCAM-immunoreactivity was localized at the cell membrane of uncommitted OE cells (PITX1^+^/LHX3^−^; **Figure 2A**) or pituitary progenitor cells (PITX1^+^/LHX3^+^; **Figure 2B**). After longer culture of organoids (day 147), many ACTH^+^ cells developed from LHX3^+^ progenitors without the loss of EpCAM expression (**Figures 2C, D**). In contrast, no EpCAM-immunoreactivity was observed for MAP2^+^ neuronal cells (**Figure 2E**). These results demonstrate that EpCAM is expressed on OE-derived pituitary lineages but not on hypothalamic NE-derived neuronal lineages in HP organoids.

It is reported that stem/progenitor cells in the developing and mature anterior pituitary of rodents consistently express Sox2 (14–17, 25, 26), a transcription factor found in different types of embryonic and adult stem cells (27). In HP organoids, SOX2-immunoreactivity was clearly observed in almost all LHX3^+^ cells on days 51 and 182 but was relatively weak or undetectable in ACTH^+^ cells (**Figures 2F, G**). Thus, HP organoids seem to maintain a stem/progenitor cell population in their EpCAM^+^ pituitary compartments.

### 3.3 Enrichment of Anterior Pituitary Cells From HP Organoids Using EpCAM

**Figure 3:**
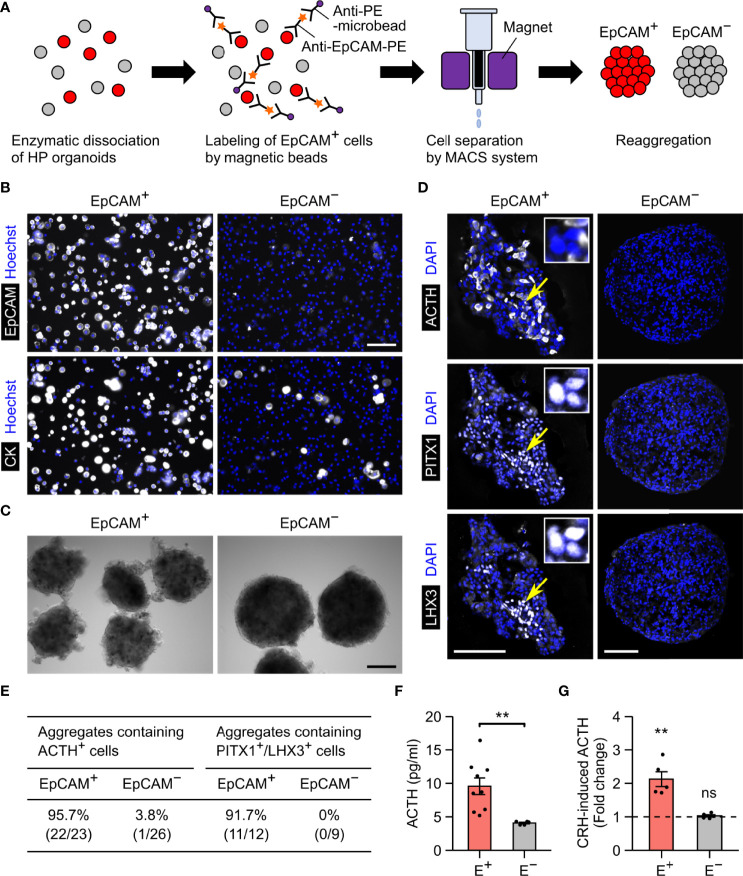
EpCAM-based sorting of anterior pituitary cells from HP organoids. **(A)** A schematic illustration of the cell sorting protocol. **(B)** Double immunostaining of MACS-separated cells for EpCAM and cytokeratin. Nuclei were stained with Hoechst. The fluorescence signals for EpCAM represent the PE labels added during the sorting process. **(C)** Representative images of EpCAM^+^ and EpCAM^−^ cell aggregates. **(D)** Triple immunostaining of an EpCAM^+^ or EpCAM^−^ aggregate for ACTH, PITX1, and LHX3. Nuclei were stained with DAPI. In the left panels, the insets show high-magnification images of three PITX1^+^/LHX3^+^ cells indicated by arrows. **(E)** Quantification of EpCAM^+^ and EpCAM^−^ aggregates containing ACTH^+^ or PITX1^+^/LHX3^+^ cells. Aggregates were obtained by at least two independent MACS experiments (see Materials and Methods). **(F)** The spontaneous secretion of ACTH from EpCAM^+^ (E^+^) and EpCAM^−^ (E^−^) aggregates. *n* = 9 (EpCAM^+^) or 5 (EpCAM^−^) experiments. **p = 0.00219 by Welch’s *t* test. **(G)** The fold change of ACTH secretion after CRH stimulation (*n* = 5 experiments). The mean value for EpCAM^+^ aggregates was significantly larger than 1 (**p = 0.00714 by one-sample *t* test), but that for EpCAM^−^ aggregates was not (ns: not significant, *p* = 0.3983 by one-sample *t* test). Scale bars: 100 µm **(B, D)**, 200 µm **(C)**.

To verify whether EpCAM is usable for purifying pituitary lineages, we separated EpCAM^+^ cells from HP organoids on days 90–500 using the MACS system (**Figure 3A**). In these experiments, the organoids were dissociated by collagenase/trypsin treatment, which did not affect the immunoreactivity of EpCAM. Prior to MACS separation, EpCAM^+^ cells constituted 32.4 ± 2.0% of the total dissociated cells; after separation, they were highly enriched in the EpCAM^+^ fraction (95.6 ± 0.7%) and reduced in the EpCAM^−^ fraction (5.6 ± 0.5%) (*n* = 3 experiments; **Figure 3B**, upper panels). As expected, cytokeratin^+^ cells were also enriched in the EpCAM^+^ fraction (**Figure 3B**, lower panels). The cell yield of the EpCAM^+^ fraction was 36.0 ± 5.0% of total dissociated cells (*n* = 4 experiments). This yield is close to the EpCAM^+^ percentage of total dissociated cells described above, indicating that the MACS system is available for isolating EpCAM^+^ cells with minimal loss.

The separated EpCAM^+^ and EpCAM^−^ cells could form aggregates (**Figure 3C**). Immunohistochemistry revealed that anterior pituitary cells (ACTH^+^ or PITX1^+^/LHX3^+^) were observed in the EpCAM^+^ aggregates but nearly absent in the EpCAM^−^ aggregates (**Figures 3D, E**). The percentages of immunopositive cells in the EpCAM^+^ aggregates were 8%–21% for ACTH, 48%–54% for PITX1, and 5%–18% for LHX3 when day-172 organoids were used for MACS (**Supplementary Figure 1**). These percentages were markedly larger than those in a previous report on unsorted HP organoids (~1% for ACTH and ~2% for LHX3 on day 100) (3) but were decreased by using older organoids for MACS (**Supplementary Figure 1**). Consistent with the immunohistochemical results, EpCAM^+^ aggregates spontaneously secreted higher levels of ACTH in comparison to EpCAM^−^ aggregates (**Figure 3F**). Furthermore, the addition of CRH led to a 2.1-fold increase in the release of ACTH from EpCAM^+^ aggregates but did not change that from EpCAM^−^ aggregates (**Figure 3G**). These results provide evidence that isolation of EpCAM^+^ cells from HP organoids enables the enrichment of anterior pituitary cells, including functional corticotrophs and stem/progenitor cells, although the efficiency of this method seems to depend on the organoid age.

## 4 Discussion

In this study, we demonstrated that EpCAM is a surface marker of anterior pituitary cells as well as their precursor OE cells in HP organoids; thus, isolation of EpCAM^+^ cells from the organoids can enrich pituitary lineages by excluding hypothalamic lineages. Using this method, we collected ~36% of organoid cells, which corresponds to ~2.8-fold enrichment of pituitary lineages. We confirmed that anterior pituitary cells were reproducibly sorted into the EpCAM^+^ cell fraction, but their proportion tended to decrease, as older organoids (>day 200) were used for the cell sorting. Although the reason for this decrease is unclear, one plausible cause is the loss of pituitary cells in the process of organoid dissociation. It was difficult to completely dissociate HP organoids cultured for >200 days, and this difficulty increased with organoid age. The undissociated cell fragments were removed before cell sorting, and we found that these fragments often contained clusters of cytokeratin^+^ cells (data not shown). Another possible cause is the downregulation of pituitary marker genes after long-term culture of organoids, a notion that would be more plausible for pituitary progenitor markers (i.e. PITX1 and LHX3).

A previous study separated OE and hypothalamic NE cells in HP organoids by fluorescence-activated cell sorting using E-cadherin, which is a surface marker for the OE but absent in the hypothalamic NE (5). In this experiment, very young organoids (day 16) were dissociated with an enzyme-free, EDTA-containing solution prior to the immunostaining for E-cadherin. In contrast, we found that dissociation of day-40 or older organoids required treatment with enzyme solutions, such as Accumax and collagenase/trypsin solutions. These enzyme treatments induced degradation of E-cadherin, which partially prevents immunostaining of OE cells for E-cadherin; only 33% of cytokeratin^+^ cells were stained for E-cadherin after Accumax treatment (data from surface marker screening). In contrast, the immunoreactivity of EpCAM was largely unaffected by enzyme treatments, thus suggesting that EpCAM surpasses E-cadherin as a surface marker for sorting OE and pituitary cells.

EpCAM is widely distributed in the epithelial tissues throughout the body (20), and its expression pattern in pituitary lineages has been reported in some studies. During mouse pituitary development, the expression of EpCAM was observed in the OE and Rathke’s pouch (the pituitary primordium derived from the OE) and then retained in pituitary stem cells (9). In postpubertal rats, EpCAM transcripts were detected in all pituitary cell types by single-cell RNA-sequencing (28). In specimens from patients with pituitary tumors, EpCAM immunostaining was found within the normal anterior pituitary and predominantly located at the cell membrane of endocrine cells (22, 23). Consistently, EpCAM was expressed on adenohypophyseal hormone-positive cells generated in monolayer culture of human ESCs/iPSCs (29). Our data compliment these previous findings: EpCAM is continuously expressed in the OE and its pituitary descendants during human pituitary development. In contrast, the expression of EpCAM was virtually absent in the hypothalamic NE and its neuronal derivatives in HP organoids. To our knowledge, this is the first report on the expression pattern of EpCAM during hypothalamic development.

We confirmed that ACTH cells separated from HP organoids retain their ability to secrete ACTH in response to extracellular CRH. We found a 2.1-fold increase in the release of ACTH from EpCAM^+^ aggregates after CRH stimulation. This effect is comparable to that observed by CRH stimulation of intact HP organoids (3), suggesting that the EpCAM-based cell sorting process has little influence on the functionality of ACTH cells. We used EpCAM^+^ aggregates derived from day-415 or day-500 HP organoids for ACTH quantification, but the spontaneous release of ACTH from HP organoids increases during days 100–300 and their responsiveness to CRH was confirmed on days 113–152 (3). These data indicate that functional corticotrophs can be collected from not only day-415 or older organoids but also younger organoids (days 100–300) through EpCAM^+^ cell sorting. In preliminary experiments, we also measured the levels of all other adenohypophyseal hormones in the culture supernatant of HP organoids, but they were very low or undetectable. We therefore did not examine the release of these hormones from EpCAM^+^ aggregates.

Our sorting method could also enrich PITX1^+^/LHX3^+^ pituitary progenitor cells. This cell group was found to express SOX2, which is considered the most common marker for pituitary stem/progenitor cells (30, 31), even after 6 months of organoid culture (**Figure 2G**). To date, much effort has been made to identify stem/progenitor cell populations in the mature pituitary gland (30). Most studies targeted rodent species and revealed that Sox2^+^ adult stem/progenitor cells are located within two types of niches: the marginal cell layer lining the pituitary cleft, and the cell clusters scattered in the anterior lobe parenchyma (16, 19, 25, 32). A similar organization of stem/progenitor cell niches was also suggested in humans (11, 31, 33, 34) and cattle (35, 36) by analyzing the expression of SOX2 and other candidate markers. Moreover, a clonogenic cell population, which expresses LHX3 and which can differentiate into prolactin^+^ cells, was isolated from normal human pituitaries from autopsies (age 51–83) (12), supporting the existence of functional stem/progenitor cells in the human pituitary gland throughout life. Recently, a single-cell transcriptomic analysis of the human fetal pituitary identified a stem cell cluster that expresses *SOX2*, *PITX1*, *LHX3*, and *EPCAM* (37). This combination of gene expression was also detected in a *Sox2*-enriched cell cluster in the anterior pituitary of postpubertal rats by single-cell RNA-sequencing (28). Although this cluster was defined as “folliculostellate cells” based on the expression of known markers, such as S100β, the vast majority of *Sox2* ^+^ cells were assigned to this cluster. It has been proposed that S100β^+^/Sox2^+^ pituitary cells serve as adult stem/progenitor cells to supply hormone-producing cell types (14, 16, 32, 38). The overlapping expression of Sox2/Lhx3/EpCAM was also found in the nestin^+^ cell group in the adult mouse anterior pituitary, and this group was capable of self-renewing and generating all six types of hormone-producing cells *in vitro* (9). Thus, we speculate that the EpCAM^+^ cell fraction from HP organoids might be a promising source of stem/progenitor cells that reside in the human pituitary gland.

Adult stem/progenitor cells have been isolated from the native pituitary gland as a clonogenic population by culturing dispersed pituitary cells in a medium containing serum and/or specific growth factors (7, 8, 12, 18, 35). In addition, such a stem/progenitor cell population can be enriched by different approaches based on its unique features: uptake of a fluorescent dipeptide (7), Hoechst dye efflux capacity (8, 10, 25), resistance to enzymatic dissociation (16), adhesion to culture-ware (19), and the expression of molecular markers, including nestin (9), GFRα2 (11), Sox2 (13, 15, 18), S100β (14), Sox9 (15), and CD9 (17, 39). Future studies should examine whether these isolation and enrichment methods are applicable to HP organoids when combined with EpCAM^+^ cell sorting.

The main limitations of our method are the technical challenges associated with the induction of HP organoids from iPSCs. First, a high-oxygen condition is required for cells to survive in organoid culture from day 18 onward. Second, long-term culture of organoids is necessary to generate functional corticotrophs. ACTH^+^ cells appear in the organoids on day 60 (3), and ACTH secretion continues to increase until day 300, as described above. Finally, there is some inter- and intra-batch variability in the efficiency of pituitary differentiation from the OE (3). This variability critically affects the proportion of pituitary lineages in sorted EpCAM^+^ cells, since EpCAM is expressed on both OE and pituitary cells. The further improvement of the differentiation efficiency of HP organoids is needed to extend the usefulness of EpCAM in enriching pituitary cells.

In conclusion, we established a novel methodology for the *in vitro* preparation of human anterior pituitary cells using a recently developed organoid technology. Human iPSCs are highly expandable in culture, amenable to genome engineering, and can be derived from patients with specific disorders. Hence, our method of purifying iPSC-derived pituitary cells would be valuable for basic and translational research on the human pituitary gland, such as stem cell biology, pathophysiology, drug discovery, and regenerative medicine.

## Data Availability Statement

The raw data supporting the conclusions of this article will be made available by the authors, without undue reservation.

## Author Contributions

YK, HS, HA, and HN contributed to conception and design of the study. YK, MK, HS, TaK, CO, MS, AK, and ST performed the experiments. YK, MK, ToK, KS, AN, and HN analyzed the data. YK, HS, and HN wrote the manuscript. All authors contributed to the article and approved the submitted version.

## Funding

This work was supported by the Japan Agency for Medical Research and Development (AMED) under Grant Numbers JP13bm0404018, JP18bm0404036, and JP21ek0109524 (to HS and HN); the Japan Society for the Promotion of Science (JSPS) KAKENHI Grant Numbers JP20K08859 (to HS), JP20K08896 (to HN), JP20K11658 (to YK), and JP20K15917 (to MK); the Japan Science and Technology Agency (JST) FOREST Program Grant Number JPMJFR200N (to HS).

## Conflict of Interest

YK, MK, HS, AK, ST, and HN are co-inventors on patent applications related to the study presented in this article. CO and HS are co-inventors on a patent involving organoid preparation. AK and ST are employed by Sumitomo Pharma Co., Ltd.

The remaining authors declare that the research was conducted in the absence of any commercial or financial relationships that could be construed as a potential conflict of interest.

## Publisher’s Note

All claims expressed in this article are solely those of the authors and do not necessarily represent those of their affiliated organizations, or those of the publisher, the editors and the reviewers. Any product that may be evaluated in this article, or claim that may be made by its manufacturer, is not guaranteed or endorsed by the publisher.

## Supplementary Material

Supplementary Figure 1 The percentage of cells immunoreactive to ACTH **(A)**, PITX1 **(B)**, or LHX3 **(C)** in the aggregates of MACS-sorted EpCAM+ and EpCAM- cells. Four independent batches of HP organoids were used for MACS, and day of sorting for each batch is presented by the table in **(A)**. The bar plots represent the mean ± SEM (n = 3-7 aggregates per batch).

## Abbreviations

ACTH, adrenocorticotropic hormone; CSM, candidate surface marker; CRH, corticotropin-releasing hormone; EpCAM, epithelial cell adhesion molecule; ESC, embryonic stem cell; gfCDM, growth factor-free chemically defined medium; HP, hypothalamic-pituitary; iPSC, induced pluripotent stem cell; KSR, knockout serum replacement; MACS, magnetic-activated cell sorting; NE, neuroepithelium; OE, oral ectoderm; RT, room temperature.
