# Supplementary material for: EpCAM Is a Surface Marker for Enriching Anterior Pituitary Cells From Human Hypothalamic-Pituitary Organoids
Source: Front Endocrinol (Lausanne). 2022 Jul 12;13:941166. doi: 10.3389/fendo.2022.941166 (PMC9316845; doi:10.3389/fendo.2022.941166)
Supplement: Supplementary Figure 1 — The percentage of cells immunoreactive to ACTH (A), PITX1 (B), or LHX3 (C) in the aggregates of MACS-sorted EpCAM+ and EpCAM- cells. Four independent batches of HP organoids were used for MACS, and day of sorting for each batch is presented by the table in (A). The bar plots represent the mean ± SEM (n = 3-7 aggregates per batch). [file Image_1.pdf]

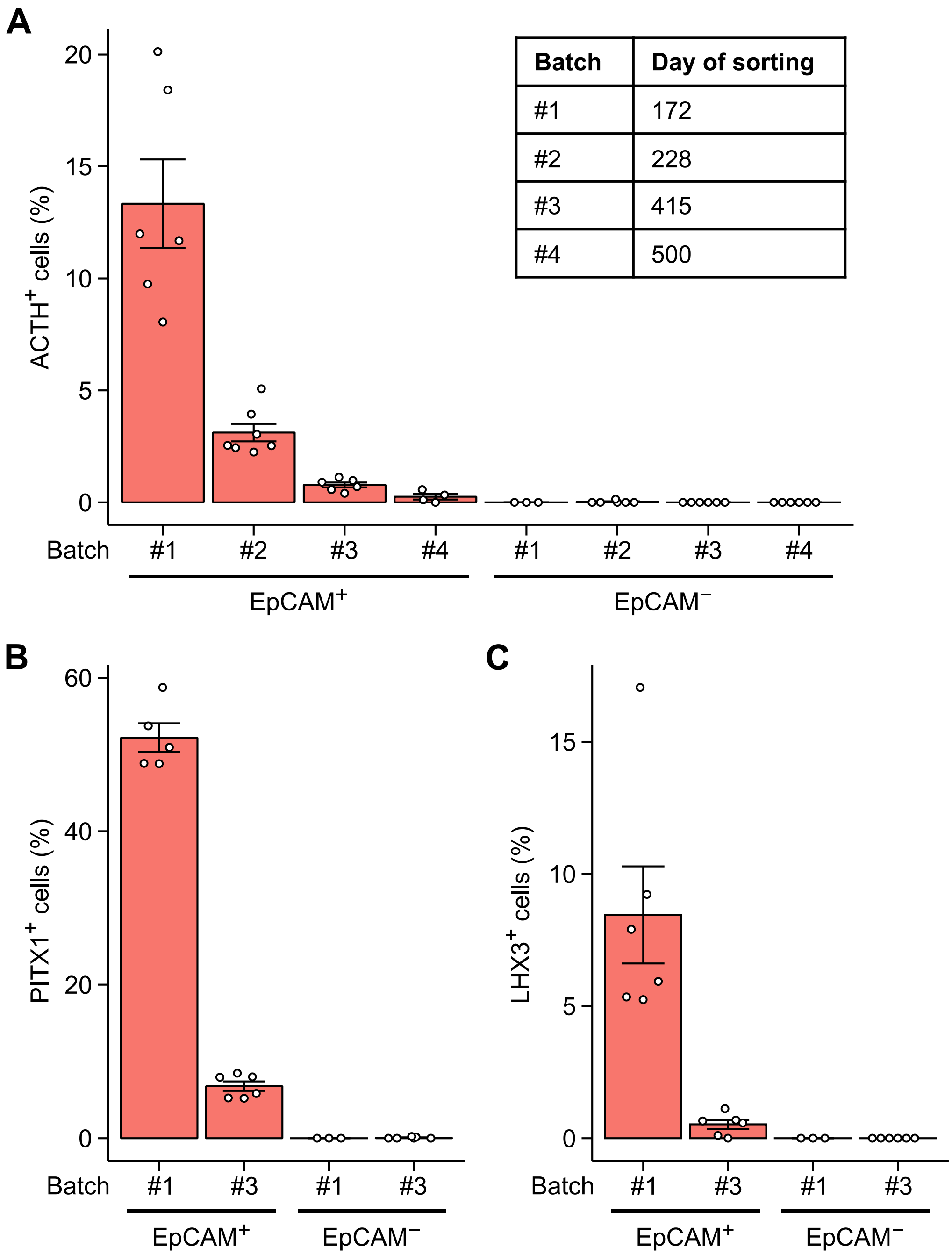

Supplementary Figure 1. The percentage of cells immunoreactive to ACTH (A), PITX1 (B), or LHX3 (C) in the aggregates of MACS-sorted EpCAM$^+$ and EpCAM$^-$ cells. Four independent batches of HP organoids were used for MACS, and day of sorting for each batch is presented by the table in (A). The bar plots represent the mean ± SEM (n = 3–7 aggregates per batch).
